# Impact of primary food allergies on the introduction of other foods amongst Canadian children and their siblings

**DOI:** 10.1186/1710-1492-10-26

**Published:** 2014-05-27

**Authors:** Mary McHenry, Wade Watson

**Affiliations:** 1 Department of Pediatrics, IWK Health Centre, 5850/5980 University Avenue, P.O. Box 9700, Halifax, Nova Scotia B3K 6R8, Canada; 2 Division of Allergy, IWK Health Centre, 5850/5980 University Avenue, P.O. Box 9700, Halifax, Nova Scotia B3K 6R8, Canada

**Keywords:** Food allergy, Siblings, Food introduction, Anxiety

## Abstract

**Background:**

Food-allergic children frequently avoid other highly allergenic foods. The NIAID 2010 guidelines state that individuals with an IgE-mediated food allergy should avoid their specific allergens and physicians should help patients to decide whether certain cross-reactive foods also should be avoided. Patients at risk for developing food allergy do not need to limit exposure to foods that may be cross-reactive with the major food allergens. The purpose of this study was to determine if parents of food-allergic children are given advice regarding introduction of allergenic foods; if these foods are avoided or delayed; if there is anxiety when introducing new foods; and if introducing other allergenic foods leads to any allergic reaction. The study also determined if there was a similar pattern seen amongst younger siblings.

**Methods:**

An online survey was administered between December 2011 and March 2012 via *Anaphylaxis Canada*’s website, available to Canadian parents and caregivers who are registered members of the organization and who have a child with a food allergy.

**Results:**

644 parents completed the online survey. 51% of families were given advice regarding the introduction of other allergenic foods. 72% were told to avoid certain foods, and 41% to delay certain foods. 58% of parents did avoid or delay other highly allergenic foods, mainly due to a fear of allergic reaction. 69% of children did not have an allergic reaction when these foods were subsequently introduced. 68% of parents felt moderate or high levels of anxiety when introducing other foods. A similar pattern was seen amongst the younger siblings.

**Conclusions:**

Canadian parents and caregivers of children with food allergies receive varied advice from health care professionals regarding the introduction of new allergenic foods, and feel moderate to high levels of anxiety. A similar pattern may be seen amongst younger siblings. While the majority of children in our study did not have an allergic reaction to a new food, a significant proportion of children did react. A more consistent approach to the advice given by health care professionals may decrease parental anxiety. Further research to support the 2010 NIAID guidelines may be necessary to clarify recommendations.

## Background

Recent studies have shown that the prevalence of a general childhood food allergy is increasing [1]. Data from this randomized cross-sectional survey in the United States studied over forty thousand children from 2009–2010 and found a food allergy prevalence of 8% which is higher than previously reported. There is evidence that having a food allergy can impact the quality of life of food allergic children and their families [2]. A lack of information provided to parents at time of diagnosis may increase anxiety and uncertainty in how to manage risk and safety for their child [3].

Concerns about food allergy and unexpected reactions to new foods may result in avoidance of other highly allergenic foods, including the eight major food allergens (milk, egg, peanut, tree nuts, soy, wheat, fish, and crustacean shellfish). Previous studies have shown that food avoidance may not be justified in all food-allergic patients [4-7]. One study found that more than 70% of food allergic children were also allergic to or were avoiding multiple foods, despite negative tests or lack of prior reactions [4]. Among patients avoiding foods because of history of reaction, positive skin test and/or positive serum specific IgE, 78% were avoiding multiple foods (on average about 3 food allergens per person). When including all foods avoided (for any reason including no evidence of allergy), the prevalence of multiple food allergies and avoidance increased to 86% (average of about 4 food allergens per person). Therefore, children may be avoiding foods despite a lack of evidence of food allergy (negative tests or lack of prior reaction).

A more recent study found that 59% of the children 10–13 years of age avoided foods due to an adverse food reaction, despite only 26% having positive specific immunoglobulin E [5]. They also found that food avoidance was related to a child’s anxiety about an adverse food reaction and appeared independent of a doctor’s diagnosis of food allergy and advice on food avoidance. Food avoidance has also been reported despite a negative food challenge and after physician advice to reintroduce food [6,7]. Sampson and Ho [8] estimated that amongst highly atopic children, more than half of children reacted to two or three foods during blinded, placebo-controlled food challenges; however only about one third of positive tests correlated with positive food challenges.

The 2010 *Guidelines for the Diagnosis and Management of Food Allergy in the United States*, published by the National Institute of Allergy and Infectious Disease (NIAID) [9] indicate that individuals with a documented IgE-mediated food allergy should avoid ingesting their specific allergens and that health care professionals should work with patients to decide whether certain cross-reactive foods also should be avoided. For patients with a known allergy to a food, the rate of clinically relevant cross-reactivity to related foods varies, with data based on limited studies. Helping the family decide whether to avoid other foods must take into consideration that skin prick or serum testing to related foods may be positive in many cases where the food may be tolerated; cross-contact among foods in preparation may be a concern; and patients may have specific food preferences.

These NIAID guidelines also include recommendations related to dietary avoidance of cross-reactive foods in at-risk patients, defined as those individuals with other atopic disorders and those with a family history of atopy. The guidelines state that “patients at risk for developing food allergy do not need to limit exposure to foods that may be cross-reactive with the eight major food allergens (milk, egg, peanut, tree nuts, soy, wheat, fish, and crustacean shellfish)”, as there is insufficient evidence to limit cross-reactive foods in a child’s diet. In addition, the NIAID also recognizes the potential for inadequate nutrition and growth if healthy foods are intentionally avoided [9].

Despite these recommendations, children may still be avoiding certain allergenic foods, regardless of negative tests or lack of prior reaction [4-7]. There are recent studies that indicate families report a lack of information at the time of diagnosis of their child’s food allergy [10], gaps in anaphylaxis management [11], and parental frustration about what physicians recommend with regards to their child’s food allergy [12]. We found no published studies in the literature that have determined whether Canadian parents receive advice about introduction of new foods amongst food allergic children and their siblings, and if and why parents choose to limit their children’s diet beyond the foods to which they are allergic.

The aim of this study was to survey Canadian families who have a child with a diagnosed food allergy, in order to determine their experience with introducing other highly allergenic foods. These other allergenic foods include the eight major food allergens (milk, egg, peanut, tree nuts, soy, wheat, fish, and crustacean shellfish) that are responsible for 90% or more of serious adverse food-induced reactions in the United States [9]. We use the term ‘primary food allergy’ to indicate the first food or foods that a child is allergic to, so as to compare with the introduction of subsequent foods. The main objective of this study was to assess whether parents of a child with a primary food allergy are given any advice regarding introduction of other highly allergenic foods, whether they decided to avoid or delay certain foods, and if there were any subsequent allergic reactions. We also determined if they experienced anxiety when introducing new foods. The secondary objective of the study was to assess the impact of the parents’ experience with food allergy on the introduction of food to the younger siblings. We studied similar outcomes with respect to any younger siblings, and whether parents were given advice, whether foods were avoided or delayed, and whether parents experienced any anxiety with the introduction of new foods.

## Methods

A cross-sectional online survey was administered to Canadian parents and caregivers who have a child with a food allergy. The study population consisted of parents and caregivers who are registered members of *Anaphylaxis Canada*, a national non-profit charity organization created to help families with severe allergies. The authors chose a national online survey in order to target a large number of participants from a diverse population of participants from across the country. *Anaphylaxis Canada* offers free membership to Canadian families, and has access to registered members via e-mail. There were approximately 7000 members of this organization at the time of the study (December 2010).

*Anaphylaxis Canada* posted a link to the online survey through their Research Section (http://www.anaphylaxis.ca). The organization also sent an e-mail to all registered members via an “e-bulletin” that included a link to the online survey. An introductory letter outlining the study and inviting parents to participate was included in the e-mail and posted on the website. The study was approved by the IWK Health Centre Research Ethics Board.

The online survey was administered via *Opinio,* a secure online server maintained by Dalhousie University. The survey was developed by the authors and consisted of 32 questions. The introduction outlined the purpose of the study including parents’ and caregivers’ experience with introducing new foods after their child has been diagnosed with a food allergy, in addition to finding out about their experience of introducing foods known to cause allergy amongst any siblings. Parents were informed that if they had more than one child with a food allergy to consider their first child who was diagnosed with a food allergy. It was indicated that answers collected would be anonymous and confidential. Parents were invited to participate after this information was provided by selecting whether they would like to participate, and consent was implied based on this selection to proceed. The survey was designed to take approximately 10 minutes to complete. A sample of the questions are provided below:

What was the first food that caused an allergic reaction in your child? (Options: milk, egg, peanut, tree nut, soy, wheat, seafood, sesame seed, other:)

Were you given any recommendations or advice regarding the introduction of other allergic foods? (Yes/No)

Were you given any advice regarding the introduction of allergic foods in the child’s siblings? (Yes/No)

No identifying information was obtained, only demographic data such as age and gender of the child. Inclusion criteria consisted of the following: subjects must be a Canadian citizen, be a parent or caregiver of a child with a diagnosed food allergy, be fluent in English, have access to a computer and have the ability to fill out an online survey. There were no exclusion criteria. Microsoft Excel^©^ and Microsoft Access^©^ were used to determine the frequency and proportion of responses to questions. Using Microsoft Access^©^ we were able to determine the number of multiple responses to a question, ie if a child had skin testing *and* serum IgE levels to aid in diagnosis of the food allergy, or if multiple foods were avoided and which ones specifically.

## Results

### Demographics

644 parents or caregivers completed the online survey. The average age of children at time of diagnosis was 21.8 months, and the average age of children at time of the administration of survey was 9.1 years (Table 1). 59% of children were male. Approximately 82% of the children were reported to be atopic (including eczema, asthma, and allergic rhinitis). 17% of children had all three atopic conditions. Approximately 26% of parents reported having a food allergy, most commonly tree nut (31%), seafood (27%) and peanut (15%).

**Table 1:** Demographic information of study group (child with first food allergy)

Male	59.9%
Female	41.1%
Average age of child at time of diagnosis	21.8 months
Average age of child at time of survey	9.1 years
Prevalence of eczema	59.8%
Prevalence of asthma	50.3%
Prevalence of allergic rhinitis	38.6%

### Part 1: first child with food allergy

**Figure 1:**
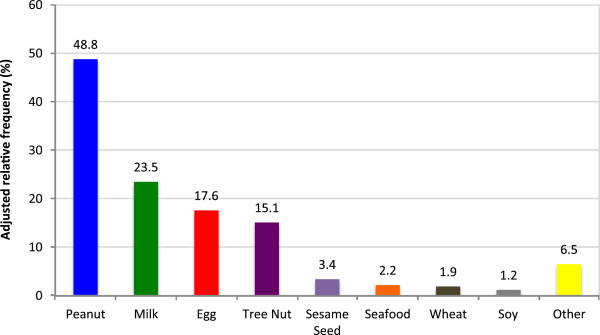
The most common food allergies in the first child with a food allergy.

The most common allergies in the first child with food allergy were peanut (48%), milk (23%), egg (17%), and tree nut (15%) (Figure 1). At the time of diagnosis, 68% of parents reported that their child had a positive skin test, 54% of parents report that a history of reaction determined diagnosis, and 24% had a positive blood test for the food. There was also a fourth option for how the food allergy was diagnosed, and 3 responders indicated that an oral challenge was performed. In addition, 82% of children had other foods tested for at the time of diagnosis, including tree nuts (84%), peanut (67%), egg (60%), seafood (53%), soy (50%), milk (49%), sesame seed (43%), wheat (40%), or other foods (20%). 85% of children were tested for multiple foods (defined as more than 1 food).

**Figure 2:**
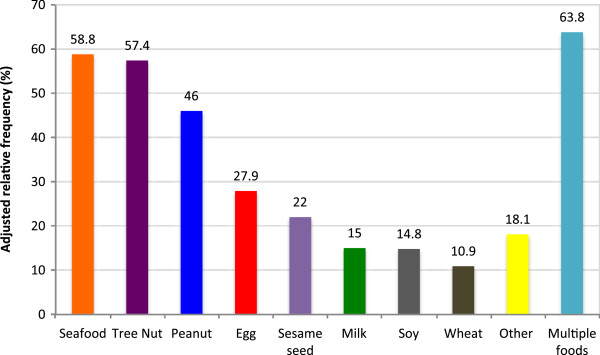
Foods that parents chose to avoid or delay introducing to their child with first food allergy.

With regards to advice given at the time of diagnosis, 51% of families reported they received advice regarding the introduction of other allergenic foods; 72% were told to avoid other allergenic foods, and 41% to delay other allergenic foods, and 14% were given other advice. This ‘other advice’ given to parents varied and included the following: introduce new highly allergenic foods individually (1%), watch closely and be cautious (1%), introduce one new food a week (0.6%), rub on skin or lip first (0.6%), introduce in small amounts (0.6%), introduce normally (0.6%). The foods parents were most commonly told to avoid or delay were tree nut (74%), peanut (64%), seafood (40%), and egg (34%). Most families (73%) were told to avoid or delay multiple foods.The vast majority (97%) of parents followed through with the advice they were provided at the time of initial diagnosis. 58% of parents did avoid or delay other highly allergenic foods, most commonly seafood (58%), tree nut (57%), peanut (46%), and egg (27%). The majority of parents did avoid or delay multiple foods (63%) (Figure 2). Parents chose to avoid or delay other foods mainly due to a fear of allergic reaction (92%). A smaller proportion (17%) reported other reasons including a positive family history (4%), to prevent food allergies (2%), concern of cross contamination (1%), concern regarding development of atopic disease (1%), and a number of other reasons. Approximately one third (31%) of children did have an allergic reaction when a new food was subsequently introduced after being initially avoided or delayed.

With regards to anxiety, 77% of parents reported that they felt anxious when introducing other foods. Most parents felt moderate (46%) or high (47%) levels of anxiety when introducing other foods.

### Part 2: experience with younger sibling

**Figure 3:**
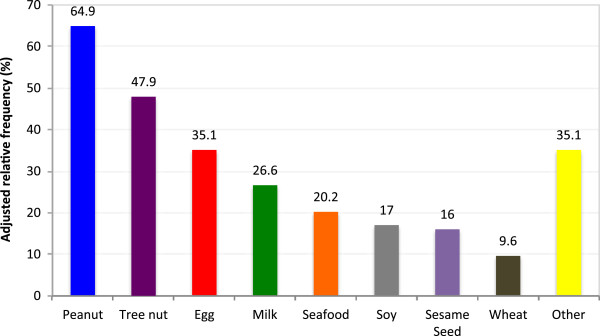
Most common food allergies amongst younger siblings.

**Figure 4:**
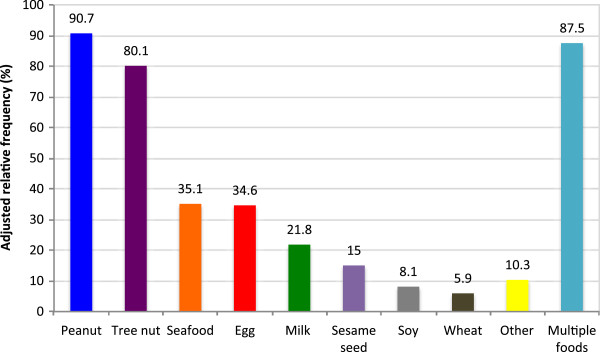
Foods that parents chose to avoid or delay introducing to the younger siblings.

The majority of children had younger siblings (57%), and 27% of these siblings had food allergies, most commonly peanut (64%), tree nut (47%), egg (35%) and milk (26%), (Figure 3). The majority (56%) of families were given advice about introducing new foods to siblings, including avoiding (35%) or delaying (67%) new foods. Parents were most commonly advised to avoid or delay peanut (86%), tree nut (70%), egg (29%), and seafood (27%). Most parents (76%) were told to avoid or delay multiple foods.Most parents (93%) reported that the knowledge of their first child’s food allergy impacted the introduction of foods to younger siblings, despite that only 27% of siblings had a food allergy. 64% of parents avoided introducing certain foods to the younger siblings, and 54% delayed certain foods. The foods most commonly delayed or avoided were peanut (90%), tree nut (80%), seafood (35%), egg (34%), and milk (21%), (Figure 4). 87% of parents avoided or delayed the introduction of multiple foods to younger siblings.

With regards to anxiety, 82% of parents report that they felt anxious when introducing foods to the younger siblings. Most parents felt moderate (49%) or high (38%) levels of anxiety when introducing new foods to younger siblings.

## Discussion

Our study highlights that the advice given to Canadian parents and caregivers regarding the introduction of new foods at the time of first diagnosis varies significantly. In our study population, approximately half of families were given advice or recommendations. The most common advice provided was to avoid or delay introducing certain foods, and most families were told to avoid multiple foods. Some families were also given other additional advice that varied greatly.

A recent study by Abdurrahman et al. [10] surveyed Canadian families about their experience with a first food allergy and identified that families report a lack of information at the time of diagnosis, specifically related to symptom recognition and management. A systematic review by Kastner et al. in [11] also highlighted gaps in anaphylaxis management, including the extent to which allergens should be excluded from a child’s diet and environment. Gupta et al. in [12] characterized food allergy knowledge, attitudes, and beliefs among parents with food-allergic children in the United States through an online survey [12]. They discovered that approximately 50% of parents felt frustrated because doctors say different things about their child’s food allergy. Hu et al. also identified that parents of children with food allergies have unmet information needs, including unaddressed topics such as what to feed their child rather than what to avoid [13]. To our knowledge, this is the first Canadian study that has looked at whether families are provided information regarding the introduction of new foods after diagnosis, and whether parents decide to avoid or delay introducing certain foods.

Our study highlights that a large proportion of parents and caregivers decide to avoid or delay the introduction of certain foods to their children and to the younger siblings. Food avoidance may have significant consequences for the child, and can have a major impact on nutrition, development and psychosocial well-being [14]. Studies have shown that food avoidance and elimination diets can result in vitamin and mineral deficiencies and negatively impact growth [15,16]. One study showed that children with cow’s milk allergy or multiple food allergies consumed less dietary calcium compared to children without cow’s milk allergy and/or only one food allergy [15]. In addition, while strict avoidance of known allergens is necessary for treatment of food allergies, delaying introduction of new foods may actually increase a child’s allergy risk topic reviewed in reference [17]. One reason for avoiding new allergenic foods may be a fear that the child will eat a cross-reactive food that could induce either sensitization or allergy to another food in the same family.

Our study also identified that while some parents and caregivers may decide to avoid or delay introducing certain foods to their child with a first food allergy, a similar pattern is seen with the introduction of food to younger siblings. In addition to the fear of cross-reactivity, parents may also wonder about introduction of allergenic foods amongst their other children. While several studies have found that some food allergic children avoid other foods despite a lack of evidence of allergy [4-7], we did not find any evidence in the literature about whether younger siblings also avoid allergenic foods. Liem at al [18] determined that siblings of peanut-allergic children are much more likely to be allergic to peanut, and recommended that these siblings be assessed by an allergist prior to feeding peanut to these children. However, the authors recognize that screening siblings prior to feeding may cause unnecessary avoidance or challenge procedures. We did not find any other studies related to the introduction of allergenic food amongst siblings of food allergic children.

It has been documented in the literature that families experience significant anxiety at the time of first diagnosis [2,3,10]. A recent study of children from a general Dutch population reported that food avoidance was related to a child’s state anxiety about an adverse food reaction, and that their doctor’s advice to avoid food did not influence food avoidance behavior [5]. Our study also highlights that families experience a significant amount of anxiety when they subsequently introduce new foods.

There is good evidence that having a food allergy can also impact the quality of life (QoL) and daily activities of food allergic children and their families [2]. Mandell et al. [3] showed that the lack of information provided to parents at time of diagnosis increases anxiety and uncertainty in how to manage risk and safety for their child, and that mothers felt inadequately supported in bearing responsibility for their children. Gillespie et al. [19] showed that amongst mothers caring for food allergic children, the feeling of “living with risk” was predominant. Once mothers understood the risks, they described an emerging feeling of “living with fear”. In addition, children who are allergic to multiple foods report a lower perceived overall health-related QoL and a greater impact on daily activities [20,21]. In our study, parents and caregivers reported a fear of allergic reaction as their main reason for delaying or avoiding foods. The majority of parents felt moderate or high levels of anxiety when introducing new foods.

While the majority of children did not react to new allergenic foods, approximately one third of children did have a subsequent reaction to a food that parents chose to initially avoid or delay. Most parents believed it was due to more than one food. Given that a significant proportion of children did subsequently react, it raises the question of whether physicians should possibly consider food challenges under observation in food-allergic children. The discussion with families regarding introducing new allergenic foods must also be considered as outlined by the NIAID guidelines [9].

We identified a number of limitations in our study. We did not determine what type of health care professional diagnosed the child with a food allergy and who provided the advice at the time of diagnosis. We recognize that there may have been a significant difference between information provided by a family doctor, pediatrician, or allergist. Our survey was not validated; however, we are unaware of any validated surveys that exist that address these specific research questions. We also did not collect any demographic information about the parents such as gender, age and level of education, which may have been important information. With respect to how the food allergy was diagnosed, we did not ask specifically if an oral food challenge was performed to aid in diagnosis, which may have helped identify which foods the patient would react to clinically.

In addition, we discovered that while the mean age of the first child with a food allergy at time of diagnosis was about 22 months, the mean age of the child at time of completion of the survey was 9 years. Thus, recall bias may have played a factor in the reporting of parent’s experiences, and possibly affected the accuracy of the information provided. Since we did not ask what year the diagnosis was made, we recognize that there may have been a difference between those children diagnosed earlier compared to more recently, as there have been changes to recommendations over the past decade. Finally, while online surveys are a reliable means of collecting data, there are potential disadvantages such as poor sample representation due to limited internet access [22]. By completing a survey through a national organization such as *Anaphylaxis Canada*, we were however, able to obtain a better representation than surveying only parents and caregivers from one city.

Future studies should determine whether there is a difference in advice regarding introduction of new foods given to families based on the type of specialist, for example between the general pediatrician, family doctor, or allergist. While our study asked families specifically about the avoidance and/or delay of new allergenic foods, it would be interesting to determine if families were told to actively introduce new foods. It may also be useful to determine which year the information was provided in order to see if the NIAID guidelines are being followed.

## Conclusions

Families of children with food allergies receive varied advice from health care professionals regarding the introduction of new allergenic foods approximately half of the time. When introducing new allergenic foods, parents and caregivers report moderate to high levels of anxiety. While the majority of children did not have an allergic reaction to a new food, a significant proportion of children did react. A more consistent approach to the advice regarding the introduction of other highly allergenic foods may decrease parental anxiety. This consistent approach may include helping families decide whether certain cross-reactive foods should be avoided and a discussion about the lack of evidence to delay highly allergenic foods in the at-risk siblings. The results of this study also raise the question of whether more oral food challenges should be performed under observation with respect to the other major food allergens these children may be avoiding, in order to confirm or exclude reactivity. Further research to support the NIAID guidelines may be necessary to clarify recommendations.

## Abbreviations

NIAID: National institute of allergy and infectious disease; QoL: Quality of life.

## Competing interests

The authors declared that they have no competing interests.

## Authors’ contributions

MM: Conceived of study; Developed online survey; Completed analysis; Wrote manuscript. WW: Conceived of study; Developed online survey; Approval of final manuscript. Both authors read and approved the final manuscript.
